# Southerners Are Wiser Than Northerners Regarding Interpersonal Conflicts in China

**DOI:** 10.3389/fpsyg.2020.00225

**Published:** 2020-02-18

**Authors:** Xin-Dong Wei, Feng-Yan Wang

**Affiliations:** ^1^Institute of Moral Education, Nanjing Normal University, Nanjing, China; ^2^School of Psychology, Nanjing Normal University, Nanjing, China

**Keywords:** collectivism, wisdom, conflict, reasoning, culture, ecology

## Abstract

Initial evidence suggests that cultural differences have consequences for wise reasoning (perspective taking, consideration of change and alternatives, intellectual humility, search for compromise, and adopting an outsider’s vantage point), with more reports of wise reasoning about interpersonal conflicts among Japanese (as compared to American) young and middle-aged adults. Similarly, we found that people from the rice-farming area of southern China also exhibited greater wise reasoning when they encountered conflicts with a friend or in the workplace than those from the wheat-farming area of northern China (*N* = 487, 25 provinces). The relationship between rice farming and wise reasoning was mediated by loyalty/nepotism. This research advances study of the relationship between wisdom and culture. It also provides evidence for the influence of social-ecological factors on wisdom and culture.

## Introduction

China has a strong sense of national identity; however, it is not immutable in culture. Research has shown that people from the traditional rice-farming regions of southern China display behaviors that are more common in interdependent cultures such as Japan. These behaviors include “holistic thought, low importance of the self, and a strong distinction between friends and strangers” (Talhelm et al., 2018, p. 1). Conversely, people who were raised in the traditional wheat-farming regions of northern China exhibit behaviors more typical of individualistic cultures such as United States, which include “analytic thought, strong importance of the self, and a smaller distinction between friends and strangers” (Talhelm et al., 2018, p. 1). These differences between the regions may cause different reasoning strategies during interpersonal conflicts in daily life. Both philosophers and psychologists have described certain reasoning strategies as wise (Baltes and Smith, 2008). Grossmann et al. (2010, 2013) presented aspects of wise reasoning that include intellectual humility, recognition of uncertainty and change, consideration of multiple ways a situation could unfold, appreciation of others’ perspectives, consideration of or search for compromise, and acknowledgment of the importance of conflict resolution. They also found that Japanese young adults showed greater use of wise reasoning during interpersonal conflicts than Americans (Grossmann et al., 2012). In this study, we asked whether people from southern China are wiser regarding interpersonal conflicts than people from northern China. When considering divorce statistics, for example, the rice-farming provinces of southern China have lower divorce rates than the wheat-farming provinces of northern China (Talhelm et al., 2014). These statistics might lead to the hypothesis that people from southern China are better at preserving intimate relationships than people from northern China and that the former may be more inclined to seek compromise with their partner than the latter. This might be construed as evidence that southerners employ wise reasoning to a greater extent than northerners.

Our research tested the hypothesis that people from the rice-farming regions in the south are more likely to use wise-reasoning strategies during conflicts with their friends or colleagues than people from the north. We also tested the mediating role of loyalty/nepotism, which is a common characteristic of rice farming and collectivist cultures (Talhelm et al., 2014; Ma et al., 2016). Loyalty/nepotism represents “the expectations of maintaining in-group harmony and mitigating interpersonal conflict whenever possible” (Wang et al., 2011, p.1298). We predicted that people from the rice-farming regions would have higher levels of loyalty/nepotism than people from the wheat-farming region, which could explain the hypothesized difference in reasoning strategies.

### Wise Reasoning: From Person-Centric Perspective to Social-Ecological Perspective on Wisdom

There are multiple ways to define wisdom (Staudinger and Glück, 2011). The person-centric approach defines wisdom as a function or characteristic of a person (Sternberg, 2019). For example, many studies have explored wisdom through autobiographic experiences of wise exemplars who were nominated by their peers (Weststrate and Glück, 2017; Weststrate et al., 2018). Moreover, scholars have also used decontextualized and global self-report scales to assess wisdom (Ardelt, 2003; Webster, 2003; Levenson et al., 2005; Glück et al., 2013). This approach has provided partial consensus concerning wisdom-related characteristics, which include openness to experiences, agreeableness, conscientiousness, extraversion, emotional stability (i.e., low neuroticism), and a greater sense of psychological and subjective well-being (Ardelt et al., 2019).

However, the person-centric perspective on wisdom has many drawbacks. Numerous well-known exemplars of wisdom (e.g., Albert Einstein, Martin Luther King, and Mahatma Gandhi) as nominated by laypeople have shown more wisdom in their professional fields than in their personal daily lives (Sternberg, 2019). This suggests that wisdom is not as stable across different situations as some personality types. Moreover, global and decontextualized self-report scales may be prone to bias, as participants tend to respond in socially desirable ways. Self-biased responding is of particular relevance when assessing wisdom, as one of the central factors of wisdom concerns intellectual humility and the absence of bias (Staudinger and Glück, 2011; Glück et al., 2013). Relying on self-report scales also inhibits scientists from exploring the processes of wisdom in everyday life (Grossmann et al., 2020). Finally, as reviewed below, increasingly many empirical studies suggest that different social-ecological contexts can significantly affect individual wisdom-related performance (Grossmann et al., 2012, 2016; Thomas and Kunzmann, 2013; Brienza and Grossmann, 2017; Zachry et al., 2018).

To overcome these drawbacks, some scholars have argued that wisdom can be better understood as a situational characteristic rather than a personal characteristic (Grossmann, 2017). They proposed characterizing wisdom from a social-ecological perspective to understand the cognitive processes underlying practical wisdom as exhibited in daily life (Grossmann et al., 2020). In this context, wisdom is best understood as wise reasoning concerning social conflicts based on the individual’s perspective-taking ability, a search for compromise and resolution, consideration of the possibility of change, and acknowledging uncertainty and the limits of one’s own knowledge (Grossmann et al., 2013). This concept predominately builds on earlier work within the Berlin Wisdom Paradigm and neo-Piagetian developmental psychology (Basseches, 1980; Baltes and Smith, 2008; Kallio, 2015). To measure wise reasoning economically using large sample sizes, Brienza et al. (2018) created the Situated Wise Reasoning Scale (SWIS), which addresses variables such as perspective taking, consideration of change and alternatives, intellectual humility, search for compromise/resolution, and adopting an outsider’s vantage point. We use this scale in the current study.

### Influence of Social-Ecological Factors on Wise Reasoning

Emerging empirical evidence indicates that social-ecological factors – such as situation, region, economics, and culture – shape the development of wise reasoning. For example, one is more likely to reason wisely when the other person involved in the situation is of higher status than oneself (Brienza and Grossmann, 2017). The same study also found that wise reasoning varies across different states of United States. Specifically, state-level affluence inversely predicts the propensity for wise reasoning. In addition to differences in wise reasoning across regions within the same culture, wise reasoning also varies among cultures. As noted earlier, Grossmann et al. (2012) found that Japanese young adults showed greater use of wise reasoning concerning interpersonal conflicts than did Americans. This suggests that the American independent culture – which promotes the view of people being unique and independent from social relationships – may inhibit wise reasoning. Compared with other proximal factors, culture as a distal factor is relatively stable and is likely to influence the elements of daily situations that individuals encounter (Oyserman et al., 2002b; Oyserman and Uskul, 2008). Therefore, it is of interest to explore wise reasoning from the perspective of this distal factor.

How people subsist within their environment plays an important role in shaping their reasoning and behavior concerning daily life events (Berry, 1967; Markus and Kitayama, 1991; Nisbett et al., 2001). Researchers compared three types of communities – farming, fishing, and herding – that share language, history, and religion in Turkey’s eastern Black Sea region and found that different eco-cultural contexts had different cognitive tendencies with regards to categorization and reasoning (Uskul et al., 2008). Similarly, different ecological contexts in southern and northern China have historically led to different social patterns. Initial evidence suggests that a history of rice farming made the people of southern China more interdependent, whereas wheat farming made the people of northern China more independent (Talhelm et al., 2014). Increasingly empirical studies suggest that historical farming can affect current-day social cognition both across China and internationally (Dong et al., 2018; Talhelm et al., 2018; Thomson et al., 2018; Liu et al., 2019). The “rice theory” – which is an extension of subsistence theory – argues that, compared with dryland crops, rice farming requires irrigation systems and approximately twice the labor hours of wheat farming, such that farmers had to share and coordinate labor more than wheat farmers (Talhelm and Oishi, 2018). These circumstances may lead to rice-farming people being likely to develop wise reasoning in social conflicts, as the social-ecological context requires maintaining harmony and mitigating interpersonal conflict.

### The Role of Loyalty/Nepotism in the Relations Between Rice Farming and Wise Reasoning

Prior research found that people from rice-farming areas of China showed more loyalty (or nepotism) toward friends over strangers (Talhelm et al., 2014). The loyalty and nepotism task was used to assess whether the participants drew a clear distinction between how they treated strangers and how they treated friends. Participants were asked to read about someone (friend or stranger) who behaved either honestly or dishonestly, which caused the participant to gain or lose certain money. They could then spend their own money to reward or punish the other person (Wang et al., 2011). The loyalty/nepotism value was obtained by subtracting the amount they punished their friend with from the amount they awarded to their friend. People with greater loyalty/nepotism are more willing to preserve relationships with honest friends and to forgive dishonest friends. When faced with conflict with a friend or a colleague in the workplace, these people may tend to take the other’s perspective and search for compromise. Bao Shuya maybe the most famous one in Chinese history. He and Guan Zhong (also known as Guanzi) were paragons of friendship, much like David and Jonathan in the Bible. They were business partners and very close companions when they were young. Guanzi invested little money in their business ventures but insisted on receiving more of the profit. When the members of Bao’s family complained, Bao simply said, “Guan Zhong’s family is poor, he needs more money than I do.” Bao also recommended Guanzi to the king, saying that Guanzi would make an excellent prime minister. Under Guanzi’s administration, Qi attained the height of its wealth and power. Later generations praise Bao’s wisdom in dealing with the relationship with Guanzi, rather than crediting Guanzi’s wise administration (Si, 2006).

Based on the information above, we proposed two hypotheses: (i) People in the rice-farming regions of southern China are wiser about interpersonal conflicts with friends or colleagues in the workplace than are people in the northern wheat-farming regions. (ii) Loyalty/nepotism explains the cultural differences in wise reasoning between southern rice farmers and northern wheat farmers.

## Materials and Methods

### Participants

We recruited 885 participants from a Chinese survey platform similar to MTurk^1^. The participants were instructed to complete “a survey of daily life” on the website. At the end of the survey, we asked participants to state where they grew up. This let us identify from which provinces they originated. To assess their attentiveness to the study while participating, we included two questions (Huynh et al., 2017). One question was the statement “I conscientiously attempted to follow instructions to the best of my ability,” which was rated on a Likert-type scale from 1 “none of the time” to 5 “all of the time.” We excluded those who indicated that they were inattentive “some of the time” or less frequently (*n* = 212). We also excluded those who were in conflict with their family members and those who indicated that the conflict started over a year ago, as they were asked to recall recent experiences in daily life and specifically with a friend or colleague (*n* = 172). Finally, we excluded participants from the historically herding provinces (Talhelm et al., 2014) and provinces that only had five members or fewer in the study group (*n* = 14; Tibet, Inner Mongolia, Xinjiang, and Hong Kong). The final sample consisted of 487 participants from 25 provinces (355 males, see Table 1 for details). Participants’ average age was 25.37 years (*SD* = 6.75). According to the website, the average time required to complete the survey was 8 min and 28 s. Each participant received 3 Yuan when they finished the survey.

**TABLE 1:** Sample size, percentage of rice-growing provinces, GDP per capita, and pathogen presence for 25 provinces.

	Number of participants (*n* = 487)	Percentage of rice	GDP per capita (10k Yuan)	Pathogen
**Rice provinces**				
Chongqing	10	0.51	0.37	NA
Fujian	13	0.81	0.67	–0.25
Guangdong	40	0.73	0.78	NA
Guangxi	11	0.59	0.35	0.61
Hubei	13	0.53	0.41	–0.50
Hunan	11	0.79	0.34	0.55
Jiangsu	33	0.60	0.73	–0.31
Jiangxi	16	0.84	0.30	0.80
Shanghai	19	0.88	1.74	–0.82
Sichuan	24	0.51	0.31	–0.04
Zhejiang	20	0.83	0.82	–0.73
**Wheat provinces**				
Anhui	23	0.33	0.33	0.77
Beijing	24	0.06	1.12	–0.64
Gansu	7	0.00	0.23	NA
Guizhou	12	0.42	0.18	1.95
Hebei	25	0.02	0.44	–0.40
Heilongjiang	6	0.10	0.54	–0.65
Henan	51	0.07	0.33	–0.14
Jilin	10	0.11	0.44	0.54
Liaoning	19	0.14	0.68	–0.46
Shaanxi	22	0.05	0.28	0.50
Shandong	40	0.02	0.57	NA
Shanxi	24	0.00	0.36	–0.24
Tianjin	7	0.11	0.98	–0.73
Yunnan	7	0.33	0.30	–0.25

*Rice-growing region data, and GDP per capita were from the 1996 Statistical Yearbook, as in Talhelm et al. (2014). Pathogen data were from a 2019 study (Liu et al., 2019).*

This research was approved by the Ethics Board of Nanjing Normal University, and the questionnaire was completed voluntarily.

### Measures

#### Wise Reasoning

The SWIS was used to measure wise reasoning ability in interpersonal conflicts (Brienza et al., 2018). Participants were first asked to recall recent experiences of conflict with a friend or colleague. To ensure the accuracy of recall, participants were asked to answer several questions about the situation and their subjective experience (for instance, “What were you doing when it happened?”; “Who was involved in this situation?”). They were then asked to respond to 21 items measuring to what extent they used the five aspects of wise reasoning: (a) consideration of others’ perspectives (“Tried to communicate with the other person what we might have in common”), (b) consideration of change and multiple ways a situation may unfold (“Looked for different solutions as the situation evolved”), (c) intellectual humility/recognition of limits of knowledge (“Double-checked whether my opinion on the situation might be incorrect”), (d) search for a compromise/conflict resolution (“Considered first whether a compromise was possible in resolving the situation”), and (e) view of the event from the viewpoint of an outsider (“Wondered what I would think if I was somebody else watching the situation”). The answers were rated on a 5-point Likert-type scale from 1 “not at all” to 5 “very much.” The Chinese version of the scale was obtained from the original authors’ website^2^. We confirmed internal consistency using Cronbach’s alpha, which was 0.90 for the present study. Scores were calculated by averaging the 21 items.

#### Loyalty and Nepotism

In this experiment, participants were asked to imagine closing a business deal with (i) an honest friend, (ii) a dishonest friend, (iii) an honest stranger, and (iv) a dishonest stranger. In the stories, the hypothetical person’s dishonesty caused the participant to lose ¥500 in the business deal, and the honesty caused the participant to gain ¥500. Participants could then spend their own money to reward (following honesty) or punish (following dishonestly) the other person. The cost was set at a tenth of the reward/punishment amount, which was presented on 11-point scales from ¥0 to ¥1,000 in ¥100 increments. The original study (Talhelm et al., 2014) found that participants from rice-farming provinces would be less likely to punish their friends than participants from wheat-farming provinces.

#### Control Variables

Prior research has found that demographic variables (social class, gender, and age) may influence participants’ wise reasoning (Grossmann et al., 2010; Brienza and Grossmann, 2017). We therefore adjusted analyses for these variables. Following previous studies (Kraus et al., 2012), we used education and family income to indicate individuals’ social class. These indicators were then standardized and collapsed into a single index.

## Results

### Zero-Order Correlations

As shown in Table 2, loyalty/nepotism was positively associated with wise reasoning (*r* = 0.31, *p* < 0.001), whereas gender (*r* = −0.08, *p* = 0.07), age (*r* = 0.07, *p* = 0.15), and social class (*r* = 0.07, *p* = 0.12) had negligible associations with wise reasoning among individuals. Among provinces, wise reasoning was positively associated with the percentage of rice farming (*r* = 0.45, *p* = 0.04) and GDP per capita (*r* = 0.46, *p* = 0.04).

**TABLE 2:** Correlations between variables at the individual and provincial levels.

Variables	*M*	*SD*	1	2	3	4	5	6
**Chinese individuals (*n* = 486)**								
1. Wise reasoning	3.38	0.72						
2. Gender (1 = male, 2 = female)	1.27	0.44	–0.08					
3. Age	25.37	6.75	0.07	–0.08				
4. Loyalty/nepotism	284.18	527.69	0.31**	–0.12	0.06			
5. Social class	0.00	1.56	0.07	0.04	0.28***	0.04		
6. Education	4.43	0.89	0.06	0.16***	0.09*	0.01	0.78***	
7. Income	5.04	2.71	0.05	−0.10*	0.35***	0.05	0.78***	0.23***
**Chinese provinces (*n* = 25)**								
1. Wise reasoning	3.37	0.27						
2. Percentage of rice	0.38	0.31	0.45*					
3. GDP per capita (10k Yuan)	0.54	0.34	0.46*	0.20				
4. Pathogen	–0.02	0.68	–0.13	0.12	−0.63**			

*p < 0.05, **p < 0.01, ***p < 0.001.

### Hierarchical Linear Model Analysis

**FIGURE 1:**
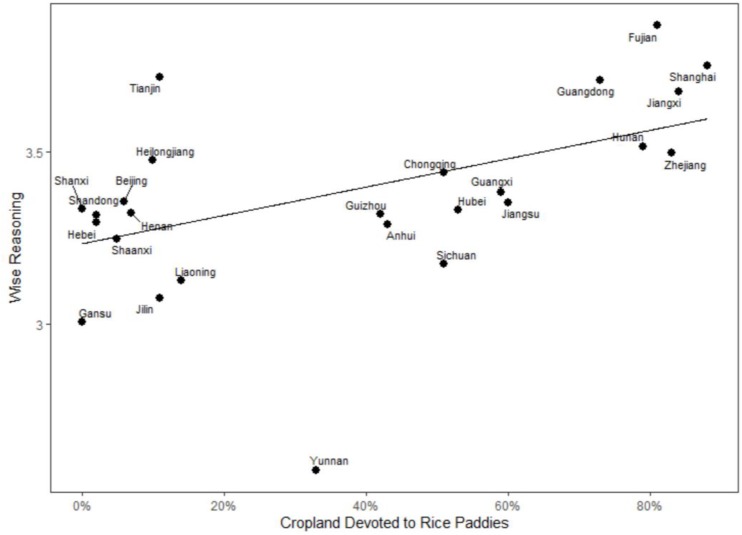
Wise reasoning by rice cultivation (Table 3, model 1).

Following the rice theory (Talhelm et al., 2014), we performed a hierarchical linear model analysis that considered participants (level 1) nested within provinces (level 2). The results in Table 3 show that rice farming predicted wise reasoning (model 1; Figure 1). The effect remained significant after we accounted for social class (model 2) and age (model 3). To disentangle the subcomponents of wise-reasoning strategies whose endorsement was best predicted by rice farming, we also ran several analyses with the subcomponents of the scale. We found that rice farming predicts all subcomponents except the outsider’s vantage point subcomponent (γ = 0.34, SE = 0.18, *P* = 0.08). The largest effect was observed for the search for compromise/resolution subcomponent (γ = 0.52, SE = 0.12, *P* < 0.001).

**TABLE 3:** Rice farming predicts wise reasoning.

		*B*/γ	SE	*t*	*p*
Model 1	Percent rice	0.41	0.11	3.90	0.001
Model 2	Percent rice	0.39	0.10	3.69	0.002
	SES	0.02	0.02	1.14	0.254
Model 3	Percent rice	0.39	0.11	3.56	0.003
	SES	0.02	0.02	0.78	0.437
	Age	0.01	0.01	0.99	0.323
Model 4	Percent rice	0.33	0.11	2.90	0.010
	SES	0.01	0.00	0.55	0.586
	Age	0.00	0.00	0.89	0.377
Modernization	Province GDP per capita	0.20	0.11	1.83	0.078
Model 5	Percent rice	0.33	0.12	2.78	0.005
	SES	0.02	0.03	0.92	0.356
	Age	0.00	0.01	0.31	0.760
Pathogen	Province pathogen prevalence	–0.05	0.06	–0.86	0.390

*We generated generalized linear mixed-models in R with the *lme4* package (Bates et al., 2015). Participants were grouped at the province level. The province GDP per capita and province pathogen data are consistent with Table 1.*

Similar to the rice-theory study, we included GDP per capita as a measure of modernization (model 4) and pathogen prevalence measures (model 5) to test modernization (Greenfield, 2016) and pathogen theory (Fincher et al., 2008). Historical GDP was used in present study since several studies have found that there’s a lag time between economic change and cultural change (Grossmann and Varnum, 2015; Thomson et al., 2018). Our study found that rice farming explained regional differences in wise reasoning, whereas modernization (γ = 0.20, SE = 0.11, *P* = 0.08) and pathogen prevalence (γ = −0.05, SE = 0.06, *P* = 0.39) did not.

### The Mediating Role of Loyalty/Nepotism

**FIGURE 2:**
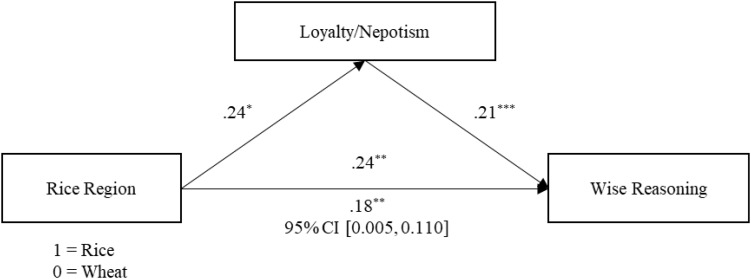
Mediation model. Values are standardized regression coefficients. The value under the rice region → wise reasoning path reveals the relationship between rice cultivation and wise reasoning after adjusting for loyalty/nepotism. The values in square brackets correspond to the 95% CIs from a bootstrap test performed to assess the significance of the indirect effect. ^∗^*p* < 0.05, ^∗∗^*p* < 0.01, ^∗∗∗^*p* < 0.001.

We subsequently examined whether loyalty/nepotism mediated the relationship between rice farming and wise reasoning using the mediation package in *R* (Tingley et al., 2015). As Figure 2 shows, rice farming was positively related to loyalty/nepotism and wise reasoning. The effect of rice farming on wise reasoning was significant after adjusting for loyalty/nepotism. Moreover, the indirect effect of rice farming on wise reasoning via loyalty/nepotism was significant (see Figure 2 for 95% confidence intervals, simulation replications = 5,000).

## Discussion

This study illuminates the differences in wise reasoning between people from southern China and northern China concerning interpersonal conflicts with friends or colleagues. People from a traditional rice-farming culture (southern China) were more likely to use wisdom-related strategies. Consistent with past research utilizing between-country comparisons (the United States vs. Japan, Grossmann et al., 2012), our results suggest that the interdependent culture of rice-farming regions – which emphasizes harmonious and stable relationships with other people – may result in wiser reasoning skills than the more independent culture in wheat-farming regions, which emphasizes uniqueness and autonomy. Our research advances the findings of Grossmann et al. (2012) in two ways. First, by analyzing differences within a single nation, this study minimized between-country alternative explanations, such as religion, language, and political influences by studying societies within the same country. Second, we found that loyalty/nepotism mediated the relationship between rice farming or collectivistic culture and wise reasoning. To some extent, this finding is consistent with past research on conflict culture in latitudinal psychology (Van de Vliert and Van Lange, 2019). Van de Vliert and Conway (2019) found that across countries in the northern hemisphere, in-group favoritism (familism from 57 countries, nepotism from 116 countries, and compatriotism from 73 countries) is stronger among southerners than among northerners. In-group favoritism – which was represented by loyalty/nepotism in our study – may encourage people to use more wisdom-related strategies such as searching for compromise and taking the other’s perspective when they face conflict with a friend or colleague. This also suggest that the relationship between loyalty/nepotism and wise reasoning might be different when people face conflict with strangers.

The results show that at the province-level, affluence was positively related to the propensity for wise reasoning, which diverges from past research (Brienza and Grossmann, 2017). Brienza and Grossmann (2017) found that middle-class ecologies promote less wise reasoning during interpersonal conflicts than do working-class ecologies across different states of United States. On average, China’s collectivistic rice-farming regions are wealthier than the wheat-farming regions. Although we included the measures of rice farming and economic affluence (GDP) in model 4 (Table 3) and found only rice farming culture explained wise reasoning, the relationship between economic resources and wise reasoning was in a positive direction (γ = 0.20, SE = 0.11, *P* = 0.08).

This divergence may result from Chinese people being more concerned about in-group harmony and having a stronger sense of duty to in-group friends than Americans (Oyserman et al., 2002a). Researchers have suggested that this primarily results from the different levels of relational mobility among countries, which refers to incentives and opportunities present in a particular environment that facilitate or hinder relationship formation and termination (San Martin et al., 2019). From the perspective of social and relational mobility (Oishi and Graham, 2010; Thomson et al., 2018), unlike upper class individuals in mobile cultures (e.g., United States), Chinese upper class individuals feel a duty to seek compromise when facing conflict with in-group members, because they cannot exit from or enter into relationships easily. They also have more resources that allow them to sacrifice their own interests, compared with their low-income friends. Therefore, we suggest that the relationship between social class and wise reasoning may be influenced by culture and friendship.

Our findings offer novel contributions to research on wisdom and culture psychology. First, our findings support the view of wisdom being a social-ecological rather than a person-centric phenomenon (Grossmann et al., 2020). Besides the effects of eastern or western culture, social class, and situational contexts (Grossmann et al., 2012; Brienza and Grossmann, 2017), we found that a traditional subsistence lifestyle can also affect wise reasoning. Second, our study supports the enduring effect of traditional subsistence lifestyle on present-day social cognition (Talhelm et al., 2014; Talhelm and Oishi, 2018). It can be safely assumed that none of our online participants had farmed for a living. However, people from rice-farming regions are wiser about interpersonal conflicts than people from wheat-farming regions, suggesting the enduring effect of rice-farming culture in present-day China.

Our study has certain limitations. First, our findings were observed using an online convenience sample, such that participants were generally younger and middle-aged adults. The generalizability of these findings to a wider age range needs to be tested, as the developmental trajectory of wise reasoning differs between collectivistic and individualistic cultures. Second, to conduct a large-scale investigation, we used a self-report scale that probed event-reconstruction of interpersonal daily experiences. Although this state-level method avoided bias because of domain-specific knowledge, the content and extent of conflicts need to be better controlled. Thus, standardized materials concerning interpersonal conflicts and observer-based evaluations would have been helpful to make our findings more robust. Third, our research focused on the mediating role of only loyalty/nepotism in the relationship between rice farming and wise reasoning without investigating other mediating roles, such as holistic/analytic thought and independent/interdependent self. In addition, the analyses are based on correlational, cross-sectional data, limiting causal inferences. Experimental and longitudinal research is thus needed to examine the causal nature of the relationships.

## Data Availability Statement

The datasets generated for this study are available on request to the corresponding author.

## Ethics Statement

The studies involving human participants were reviewed and approved by Ethics Board of the Nanjing Normal University. The ethics committee waived the requirement of written informed consent for participation.

## Author Contributions

F-YW and X-DW designed the research. X-DW carried out the research, analyzed the data, and wrote the manuscript.

## Conflict of Interest

The authors declare that the research was conducted in the absence of any commercial or financial relationships that could be construed as a potential conflict of interest.

## Footnotes

1 https://wj.qq.com

2 https://uwaterloo.ca/wisdom-and-culture-lab/measures
